# Anticancer Effects of Dihydroartemisinin on Human Esophageal Cancer Cells In Vivo

**DOI:** 10.1155/2018/8759745

**Published:** 2018-05-17

**Authors:** Cailing Jiang, Shumin Li, Yanjing Li, Yuxian Bai

**Affiliations:** ^1^The Oncology Department, The Affiliated Hospital of Guilin Medical University, Guilin 541000, China; ^2^Department of Gastrointestinal Oncology, Harbin Medical University Cancer Hospital, Harbin, Heilongjiang Province, China

## Abstract

Despite recent advances in chemotherapy and surgical resection, the 5-year survival rate of esophageal cancer still remains at the low level. Therefore, it is very important to discover a new agent to improve the life expectancy of patients with esophageal cancer. Dihydroartemisinin (DHA), a semisynthetic derivative of artemisinin, has recently exhibited promising anticancer activity against various cancer cells. But so far, the specific mechanism remains unclear. We have previously demonstrated that DHA reduced viability of esophageal cancer cells in a dose-dependent manner in vitro and induced cell cycle arrest and apoptosis. Here, we extended our study to further observe the efficacy of DHA on esophageal cancer cells in vivo. In the present study, for the first time, we found that DHA significantly inhibits cell proliferation in xenografted tumor compared with the control. The mechanism was that DHA induced cell apoptosis in both human esophageal cancer cell lines Eca109 and Ec9706 in vivo in a dose-dependent manner. The results suggested that DHA was a promising agent against esophageal cancer in the clinical treatment.

## 1. Introduction

Esophageal cancer is currently the fourth most common cause of cancer death in China. The average death attains around 150 thousand people every year [1–3]. Despite recent advances in chemotherapy and surgical resection, the 5-year survival rate remains at the low level [4, 5]. The low survival rate might be due to the lack of early diagnosis, invasion and metastasis of the tumor, and over-reliant on confined drugs with some side effects and resistance, thus producing unsatisfactory results. Therefore, it is very important to discover a new agent to improve the life expectancy of patients with esophageal cancer.

Dihydroartemisinin (DHA), a front-line antimalarial herb compound, has shown promising anticancer activity in vitro and in vivo with low toxicity to normal cells [6]. There is growing evidence that DHA inhibits the growth of various cancer cells such as cervical cancer cells [7], pancreatic cancer cells [8], prostate cancer cells [9], hepatoma cells [10], and neuroglioma cells [11]. Moreover, DHA also has suppressive effects on the invasion and metastasis of cancer cells [12].

We have demonstrated that DHA reduced the viability of esophageal cancer cells in a dose-dependent manner. The mechanism was at least partially due to DHA which induced apoptosis by upregulating the expression of Bax, downregulating Bcl-2, Bcl-xL, and procaspase-3, and increasing caspase-9 activation, which induced cell cycle arrest by downregulating cyclin E, CDK2, and CDK4. We concluded DHA might be a novel agent against esophageal cancer [13, 14]. However, whether DHA is also active against esophageal cancer cells in vivo still needs to be investigated.

In our study, we established human esophageal tumor xenografts in BALB/c nude mice and treated them with DHA in vivo and the changes of tumor size were closely monitored. We assessed its cytotoxic effects with tumor growth curves, HE staining, Ki-67 proliferation index, and TUNEL staining.

## 2. Materials and Methods

### 2.1. Ethics Statement

The research protocol was in accordance with the Institutional Guidelines of Animal Care and Use Committee at Harbin Medical University.

### 2.2. Cells and Mice

The human esophageal cancer cell lines Eca109 and Ec9706 (Laboratory of Medical Genetics, Department of Biology, Harbin Medical University) were routinely cultured in RPMI 1640 medium supplemented with 10% fetal bovine serum, penicillin (100 U/mL), and streptomycin (100 mg/mL) with 5% CO_2_ at 37°C in a humidified incubator. Animal experiments were conducted in accordance with the Guide for Care and Use of Laboratory Animals. Male mice are more aggressive than female mice. To avoid the accidental injury, female mice are chosen in this experiment. Female BALB/c nude mice, 3-4 weeks old, were obtained from the Animal Research Center (Slac, Shanghai, China). All mice were housed and maintained under specific pathogen-free conditions.

### 2.3. Reagents

DHA was purchased from Sigma-Aldrich (St. Louis, Missouri, USA) and was dissolved to 160 mmol/L in dimethyl sulphoxide (DMSO) before experiments. The anti-Ki-67 antibody was purchased from Abcam (Cambridge, Massachusetts, USA). The TUNEL agent was purchased from Roche (Shanghai, China).

### 2.4. Experimental Design

Tumors were established by subcutaneous injection of 5 × 10^6^ Eca109 or Ec9706 cells into the flanks of mice, and the success rate was 80%. Tumors with Eca109 were more stable, and tumor with Ec9706 developed more quickly. At the seventh day, tumors reached around 25 in the Eca109 group and 75 mm^3^ in the Ec9706 group; the mice of both groups were randomly assigned to four groups (4 in each group). The mice in the control group received a daily intraperitoneal injection of 25 *μ*L of DMSO, and the mice in other three treatment groups received an intraperitoneal injection of DHA at doses of 2, 10, or 50 mg/kg, respectively. The mice were closely monitored. Tumor size was measured every other day on Eca109 and every other two days on Ec9706 with calipers from the intraperitoneal injection, and tumor volume was calculated according to the formula *π*/6 × largest diameter × (smallest diameter)^2^. The mice were euthanized at about 2 weeks later according to treatment time, as the rate of liquefactive necrosis in the tumor may increase after 2 weeks, and the tumors were removed and fixed with 10% paraformaldehyde.

### 2.5. Immunohistochemistry

The expression of Ki-67 in the tumor area was detected as soon as the tumors were fixed well by the immunohistochemical method using a commercial kit according to the manufacturer's instructions. Briefly, paraffin-embedded sections were incubated overnight with an anti-Ki-67 Ab after microwave antigen retrieval. They were subsequently incubated for 30 min with the appropriate secondary Ab using the ultrasensitive TMS-P kit and immune reactivity developed with Sigma FAST DAB (3,30-diaminobenzidine tetrahydrochloride). Sections were counterstained with hematoxylin, mounted, and examined by microscopy. Appropriate positive and negative controls were run with each batch.

### 2.6. In Situ Detection of Apoptotic Cells

Apoptotic cells were detected by TUNEL using the ApopTag Peroxidase in Situ Cell Apoptosis Detection Kit (Roche, UK), according to the manufacturer's instructions. Per tumor section above was covered with TUNEL mixture (2 *μ*L TdT, 48 *μ*L Lable solution) and 50 *μ*L additional converter POD after dewaxing and a 30 min incubation at 15–25°C with 3% BSA, 20% normal bovine serum in Tris-HCl, and 0.1 M Ph. A TUNEL negative control was obtained by omitting TdT from the labeling mix, and TUNEL specificity was checked by comparing labeling with cellular morphology.

### 2.7. Statistical Analysis

For the analysis of immunohistochemistry for Ki-67 cells and apoptotic cells, at least two randomly selected images chosen randomly at ×200 magnification figure were used. The area and mean fluorescence intensity of positive staining (integrated optical density (IOD)) were determined as previously described within a defined fluorescence intensity threshold applied to all sections and analyzed using Image Pro Plus software [15]. The IOD results were expressed per total area of the given section, and the IOD values of an experimental animal were averaged. All results were presented as the mean values ± SD (standard deviation). The data were compared with the SNK (Student-Newman-Keuls) analysis and analysis of variance (ANOVA), and *P* < 0.05 was considered statistically significant.

## 3. Results

### 3.1. Tumor Volume Change

**Figure 1:**
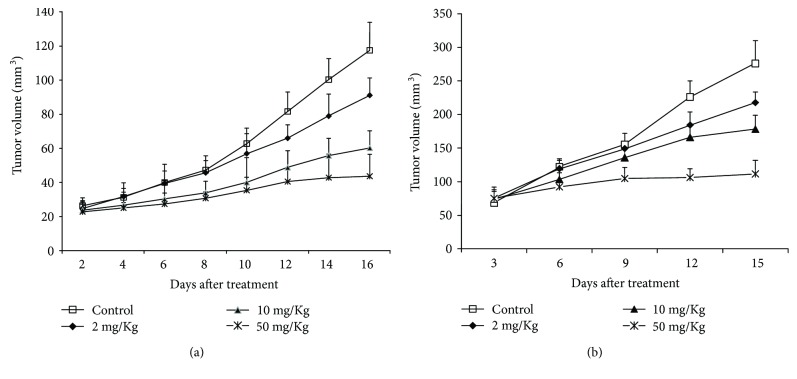
The measure of tumor volume of Eca109 and Ec9706. (a) In the non-DHA-treated control group, tumor volume increased remarkably quickly. Compared with the control group, the tumor growth was significantly suppressed by 22.9%, 48.9%, and 63% in Eca109 in mice treated with 2, 10, and 50 mg/kg DHA (*P* < 0.05). (b) In the non-DHA-treated control group, tumor volume increased remarkably quickly. Compared with the control group, the tumor growth was significantly suppressed by 21.1%, 35.3%, and 60% in Eca9706 in mice treated with 2, 10, and 50 mg/kg DHA (*P* < 0.05).

When the tumors reached a volume of around 25 mm^3^ in Eca109 and 75 mm^3^ in Ec9706, the mice were randomly assigned to four groups to receive an injection of one thousand DMSO (control), 2, 10, or 50 mg/kg DHA, respectively (as shown in Figure 1). In the non-DHA-treated control group, tumor volume increased remarkably quickly. A significant difference in tumor volume was observed between the non-DHA-treated control and the DHA-treated groups on day 8; in addition, statistically significant differences in tumor volume were noted among the three treated groups on day 10 after treatment. Compared with the control group, the tumor growth was significantly suppressed by 22.9%, 48.9%, and 63% in Eca109 (*P* < 0.05), while 21.1%, 35.3%, and 60% in Eca9706 (*P* < 0.05) in mice treated with 2, 10, and 50 mg/kg DHA.

### 3.2. Dihydroartemisinin Inhibits Cell Proliferation In Situ

**Figure 2:**
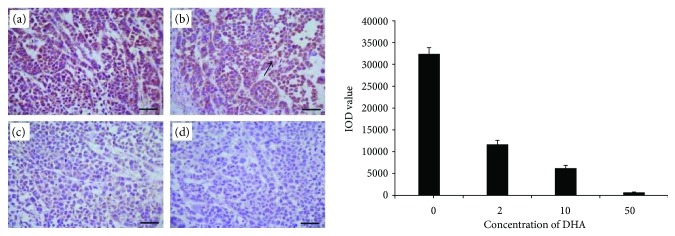
Examination of Ki-67 immunostaining in Eca109. The IOD value remarkably decreased in the 2 mg/kg DHA group (b) (11676.1 ± 924), 10 mg/kg DHA group (c) (6239.8 ± 624), and 50 mg/kg DHA group (d) (687.6 ± 106.6) when compared with the value in the control group (a) (32395.6 ± 1427.2; *P* < 0.05). The arrow points to the Ki-67-positive cell. Scale bars: 50 *μ*m.

**Figure 3:**
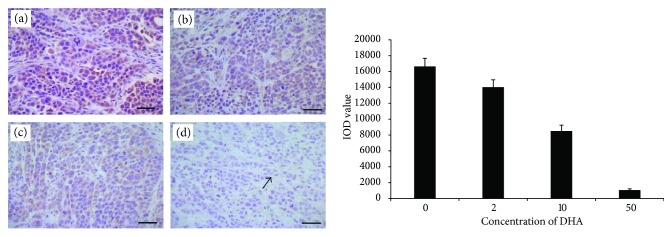
Examination of Ki-67 immunostaining in Eca9706. The IOD value remarkably decreased in the 2 mg/kg DHA group (b) (14,031 ± 939.3), 10 mg/kg DHA group (c) (8508.6 ± 736), and 50 mg/kg DHA group (d) (1053.2 ± 172.7) when compared with the value in the control group (a) (16642.7 ± 1023.1; *P* < 0.05). The arrow points to the Ki-67-positive cell. Scale bars: 50 *μ*m.

Immunostaining for Ki-67 was exclusively nuclear. Ki-67 expression was significantly related to increased tumor cell proliferation. Elevated Ki-67 expression was associated with a high mitotic index and high histological grade; based on that, we showed DHA inhibited the proliferation of Eca109 and Ec9706 cells in situ. In our results, we have examined Ki-67 staining and assessed the IOD values for Ki-67 staining. In Eca109, the IOD value remarkably decreased in the 2 mg/kg DHA group (11676.1 ± 924), 10 mg/kg DHA group (6239.8 ± 624), and 50 mg/kg DHA group (687.6 ± 106.6) when compared with the value in the control group (32395.6 ± 1427.2; *P* < 0.05). In Ec9706, the IOD value remarkably decreased in the 2 mg/kg DHA group (14031 ± 939.3), 10 mg/kg DHA group (8508.6 ± 736), and 50 mg/kg DHA group (1053.2 ± 172.7) when compared with the value in the control group (16642.7 ± 1023.1; *P* < 0.05, Figures 2 and 3). Additionally, the inhibitory effects of DHA therapy on cell proliferation displayed a dose-dependent manner, as Ki-67-positive cells in the 10 mg/kg DHA group (Eca109: 6239.8 ± 624, Ec9706: 8508.6 ± 736) were significantly fewer than those in the 2 mg/kg DHA group (Eca109: 11676.1 ± 924, Ec9706: 14031 ± 939.3), and even fewer Ki-67-positive cells in the 50 mg/kg group (Eca109: 687.6 ± 106.6, Ec9706: 1053.2 ± 172.7) than in the 10 mg/kg group.

### 3.3. Dihydroartemisinin Induces Cell Apoptosis In Situ

**Figure 4:**
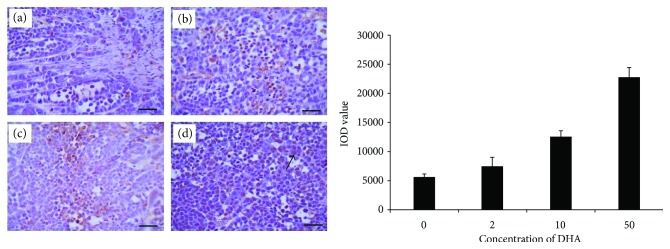
Detection of TUNEL staining in Eca109. The IOD value remarkably increased in the 2 mg/kg DHA group (b) (8010.9 ± 1477), 10 mg/kg DHA group (c) (11643.7 ± 744.4), and 50 mg/kg DHA group (d) (22555.5 ± 1542.9) when compared with the value in the control group (a) (5617.7 ± 408; *P* < 0.05). The arrow points to the TUNEL-positive cell. Scale bars 50 *μ*m.

**Figure 5:**
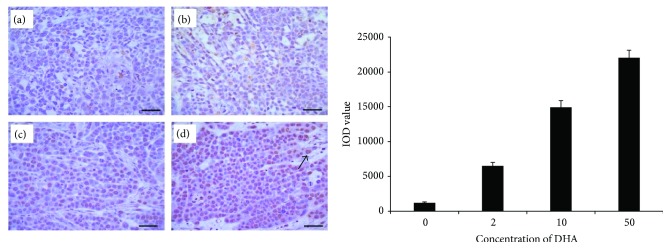
Detection of TUNEL staining in Eca9706. The IOD value remarkably increased in the 2 mg/kg DHA group (b) (6515.8 ± 507), 10 mg/kg DHA group (c) (14915.2 ± 972.1), and 50 mg/kg DHA group (d) (22019.4 ± 1093.1) when compared with the value in the control group (a) (1209.5 ± 118.4; *P* < 0.05). The arrow points to the TUNEL-positive cell. Scale bars 50 *μ*m.

TUNEL staining was applied to detect the apoptosis of tumor cells in the four groups. We assessed the IOD values for TUNEL staining. In Eca109, the IOD value remarkably increased in the 2 mg/kg DHA group (8010.9 ± 1477), 10 mg/kg DHA group (11643.7 ± 744.4), and 50 mg/kg DHA group (22555.5 ± 1542.9) when compared with the value in the control group (5617.7 ± 408; *P* < 0.05). In Ec9706, the IOD value remarkably increased in the 2 mg/kg DHA group (6515.8 ± 507), 10 mg/kg DHA group (14915.2 ± 972.1), and 50 mg/kg DHA group (22019.4 ± 1093.1) when compared with the value in the control group (1209.5 ± 118.4; *P* < 0.05). The large amount of apoptosis may be due to the presence of DHA, which was in line with the result of Ki-67 staining. TUNEL staining had a positive correlation with variable doses of showing a dose-dependent manner (Figures 4 and 5).

## 4. Discussion

In the current study, we firstly report the antitumor effect of DHA on esophageal cancer in vivo. DHA-treated cells showed characteristics of inhibited growth and promoted apoptosis, suggesting that DHA might be a potent and promising agent to combat esophageal cancer. The antiproliferation effect of DHA in many types of cells has been previously shown [7–12]. Consistent with these reports, our results showed that DHA exerted cytotoxicity on esophageal cancer cells. DHA inhibited cell proliferation and viability in a dose-dependent manner. Because main hallmarks of cancers result from the uncontrolled cell cycle and evading apoptosis [16], therapeutic drugs such as DHA, which can arrest the cell cycle and promote apoptosis in cancer cells, are highly desired.

DHA, as a novel and promising drug for cancer, exhibits anticancer activity in various cancer cells [7–12], but the effect of DHA on esophageal cancer remains incompletely understood, and our previous study demonstrated that DHA might be a novel agent against esophageal cancer in vitro, the data for which is already published, supporting the theory that DHA possesses the underlying mechanisms against esophageal cancer [13]. Here, the absence of data in vivo was remedied.

Ki-67, a commonly accepted nuclear protein, is associated with cell proliferation in many malignant tumors. As a valuable addition to experimental study and the histopathological assessment of some cancers, Ki-67 provides important prognostic information to support prognostic evaluation in the treatment [17–19]. Wiesner et al. demonstrated that it is statistically significant for the overall survival by examining Ki-67 as a prognostic marker in routine clinical use in breast cancer patients [20]. In addition, Klintman et al. recommended that as a prognostic tool, Ki-67 could serve as an alternative or complement factor for cancer progression to histological grade [21]. As a result, the Ki-67 index is one of the most important markers of proliferation in tumors.

In our study, Ki-67 immunohistochemistry demonstrated that DHA had reduced cell proliferation. Similar to our study, some experts found that DHA induced hyperexpression of Ki-67 in pancreatic cancer cell and ovarian cancer cell [22, 23]. Our data showed that there was a correlation between the inhibition of nuclear reactivity and the presence of DHA and the extent of nuclei stained was highly correlated to the concentration of DHA. The positive nuclei were more abundant in the control group than in the treated groups. We deduced that DHA might inhibit the growth of tumors and improve survival by impeding cell proliferation. Consistent with our results, to examine DHA as a therapeutic drug on breast cancer cells, Lucibello et al. concluded that DHA inhibited cell proliferation and induces apoptosis by targeting the phosphorylated form of TCTP [24]. Similarly, Feng et al. have found that treatment with cisplatin combined with DHA could enhance cisplatin-induced proliferation inhibition in ovarian cancer cells. This mechanism is at least partially due to DHA deactivation of mTOR kinase and promotion of apoptosis [25]. Furthermore, in a recent study, Odaka et al. analyzed the anticancer activity of DHA in rhabdomyosarcoma cells; the authors came to the conclusion that DHA might represent a novel class of inhibitor by meditating the mammalian target of rapamycin (mTOR), a central controller for cell proliferation and survival in the tumor cells [26].

Apoptosis, which differs from necrosis, is an effective, noninflammatory way to remove redundant or damaged cells from tissues thereby securing tissue homeostasis [27]. At present, two main caspase pathways have been identified, the mitochondrial apoptosome-mediated intrinsic pathway or the death receptor-induced extrinsic pathway. Both pathways are interconnected at several levels. The most important protein regulating apoptosis is Bcl-2, which is located on the membrane of the mitochondria, whereas Bax directly binds to Bcl-2 and inhibits its function [27]. An increase in the ratio of Bax/Bcl-2 stimulates the release of cytochrome c from the mitochondria into the cytosol, and the cytosolic cytochrome c then binds to proapoptotic protease activating factor-1 (Apaf-1), resulting in the activation of an initiator caspase which starts proteolytic events that kill the cell [28–30].

The results of the present study showed that the induction of the apoptosis of cancer cells might be an important mechanism for DHA on tumor cell viability [31, 32]. In the case of pancreatic cancer, Chen et al. suggested that DHA induced apoptosis by reducing the ratio of Bcl-2/Bax and increasing the activation of caspase-9 in a dose-dependent manner [22]. Similarly, Zhang et al. revealed that DHA induced apoptosis, activated caspase-3, and increased the ratio of Bax/Bcl-2 in human hepatoma cells [10]. What is more, Jiao et al. showed that DHA induced apoptosis in human ovarian cancer cells with a decrease of Bcl-xL and Bcl-2 and an increase of Bax and Bad [23]. These findings indicated that DHA exhibited anticancer activity partly by apoptosis.

As detailed in our previous paper [13], DHA treatment induced a significant apoptosis in the two types of esophageal cancer cells in a dose-dependent manner. Our present TUNEL results indicated that the apoptosis rate of esophageal cancer cells in DHA treatment groups was higher than that in the control group, while the apoptosis rate of esophageal cancer cell in the 2 mg/kg DHA group was apparently lower than that in the 50 mg/kg DHA group. These outcomes have suggested that an increased concentration of DHA may lead to increased cell apoptosis or death. Similar to our study, Xu et al. demonstrated that DHA facilitated apoptosis in prostate cancer PC3 cells [9].

In conclusion, we investigated the anticancer effects of DHA on human esophageal cancer cells in vivo. This study suggested that DHA inhibited cell proliferation and induced apoptosis in esophageal cancer, and we did not observe adverse effects. Our study indicated that DHA was a very interesting natural product, and we wish a bright future to it. We will process more histological and molecular studies to promote the translation of DHA to the clinic.
